# Documentation of antipsychotic-related adverse drug reactions: An educational intervention

**DOI:** 10.4102/sajpsychiatry.v25i0.1378

**Published:** 2019-11-27

**Authors:** Gregory Purcell, Jane McCartney, Shirley-Anne Boschmans

**Affiliations:** 1 Department of Pharmacy, Faculty of Health Sciences, Nelson Mandela University, Port Elizabeth, South Africa; 2 School of Pharmacy, Faculty of Natural Sciences, University of the Western Cape, Cape Town, South Africa; 3 Private, Johannesburg, South Africa

**Keywords:** adverse drug reactions, antipsychotics, documentation, educational intervention, clinical audit

## Abstract

**Background:**

Antipsychotic agents are associated with harmful adverse reactions which impact negatively on patient adherence and clinical management. Accurate and complete documentation of signs and symptoms in the clinical notes is an important means of communication between healthcare providers, and an essential component in the management of antipsychotic-induced adverse drug reactions.

**Aim:**

To determine the impact of an educational intervention on the incidence and extent of antipsychotic-induced adverse drug reaction documentation in patient medical records.

**Setting:**

The research was conducted in an acute care, public sector psychiatric facility in the Eastern Cape province of South Africa.

**Methods:**

A quasi-experimental, before and after method was used, which focused on an educational intervention. The study design consisted of three phases: pre-intervention, intervention and post-intervention. A clinical audit was conducted, reviewing 102 patient medical records in the pre-intervention phase and a further 102 patient medical records in the post-intervention phase, in order to determine the impact of the intervention on the frequency and extent of documentation of suspected antipsychotic-induced adverse drug reactions

**Results:**

Following the educational intervention, documentation of adverse drug reactions to antipsychotic drugs increased from 66 instances in the pre-intervention phase to 82 instances in the post-intervention phase. A statistically significant increase (Pearson’s Chi-square *p* < 0.05) was observed in the number of patient medical records that identified suspected adverse drug reactions.

**Conclusion:**

The educational intervention was found to increase the incidence of documentation of adverse drug reactions, and increased awareness of the potential adverse drug reactions associated with antipsychotic drugs following the intervention.

## Introduction

Antipsychotic drugs have been in use since the 1950s and form the basis of treatment for psychotic disorders.^1^ The antipsychotics are used to treat a variety of psychiatric disorders, including schizophrenia, schizoaffective and delusional disorders, as well as conditions such as bipolar mood disorder, major depressive disorders with psychosis, psychotic disorders linked to acute substance abuse and dementia.^2,3,4,5^ Although antipsychotic drugs are often combined with non-pharmacological interventions, in some cases these drugs may be the only effective treatment available, such as when treating floridly psychotic patients.^6,7^

Despite the proven value of antipsychotic agents, there are problems associated with their use, foremost of which is the risk of adverse drug reactions (ADRs).^5,8,9^ The numerous adverse effects associated with antipsychotic use may include dry mouth and sedation, impaired cognition and extrapyramidal side effects (EPSE), adverse metabolic effects such as hyperprolactinemia, impaired glucose and lipid metabolism, weight gain, cardiac effects like QTc prolongation and postural hypotension, and sexual dysfunction.^10^

In the USA, the incidence of serious ADRs requiring hospitalisation was found to be 6.7%, whilst 10.9% of inpatients were estimated to experience an ADR during hospitalisation.^11^ ADRs often prolong the hospital stay for patients, raising individual costs per patient.^11,12^ A meta-analysis identified that nearly half of all ADRs in adult outpatients and inpatients could have been prevented.^13^ In South Africa, a cross-sectional study found that 8.4% of admissions to adult medical wards in four public sector hospitals across three different provinces were directly attributable to ADRs.^14^ Numerous studies focusing on antipsychotic drugs have demonstrated that antipsychotic-induced ADRs have a negative impact on patient adherence to medication, both in an institutional setting and post-discharge, in the outpatient setting.^15,16,17^ Thus, it is in the best interest of both the patient and the clinical team to reduce ADRs and to prevent recurrence.

Reduction of ADRs as a means of enhancing patient safety requires an effective management strategy, which should include accurate documentation and reporting in the clinical notes.^18^ Documentation requires the recording of details of any suspected adverse drug reaction, as well as clinical symptoms and observations in the clinical notes. Proper documentation of ADRs is essential for the prevention of avoidable reoccurrence of previously experienced ADRs and can be used to guide therapeutic decisions in a way that avoids exposing the patient to risk of similar ADRs.^13^

To further emphasise the usefulness of documentation, when managing ADRs one must consider the typical institutional setting. Nurses have greater exposure to patients and, therefore, need to ensure accurate and complete documentation of ADRs in order to communicate the potential risk to the prescriber, who is ultimately responsible for the management of the observed or suspected ADRs. Accurate, complete and relevant documentation, therefore, facilitates communication between the nurse, patient and prescribers, and if documentation is lacking or inadequate, discontinuity of care occurs as a result of the impaired communication.^19^ Several studies have reported deficiencies in the quality of nursing documentation with inaccurate or inadequate patient data,^20^ insufficient reporting of clinical signs and symptoms^21^ and little mention of the patients’ self-identified needs and symptoms.^22^ However, if documentation is viewed as an essential communication tool, then the accuracy and completeness of the clinical notes cannot be the sole task of the nursing staff, as the responsibility for patient safety must be shared by all healthcare professionals (HCPs) involved with inpatient admissions. In Australia, an audit of the clinical notes of psychiatric inpatient admissions focused on the documentation of medication changes by prescribers and identified substantial gaps in essential clinical information. The authors highlighted that the failure to communicate effectively through the documentation process in the clinical notes put HCPs, patients and the institutions at risk.^23^

Several interventions have been attempted internationally to improve documenting and reporting of ADRs, with reasonable success.^23,24,25,26^ The advent of electronic ADR reporting systems was found to increase interdisciplinary involvement in documenting and reporting ADRs and improved communication between HCPs.^24^ Although the transition from paper-based records to electronic health records has been implemented in many countries, with associated reductions in the incidence of ADRs having been reported,^25^ paper-based patient medical records are still widely used in South Africa’s public healthcare sector. In the South African context, there is a lack of information in the published literature regarding the incidence of antipsychotic-induced ADRs, whilst no research appears to have been conducted on the level of ADR documentation in the clinical records.

## Research aim and objectives

This study aimed to evaluate the effectiveness of an intervention aimed at improving the documentation of antipsychotic-induced ADRs in a public sector acute psychiatric facility in the Eastern Cape. The research aim was achieved by meeting the objectives of (1) assessing pre-intervention practice at the site, (2) implementing the intervention and (3) assessing post-intervention practice. An educational intervention was chosen over the option of simply introducing a new method of documentation, in order to acquaint HCPs with the documentation process in a hands-on manner, and to remedy the perceived lack of insight into the seriousness of the antipsychotic-induced ADRs.

## Research methodology

### Study design

**FIGURE 1:**
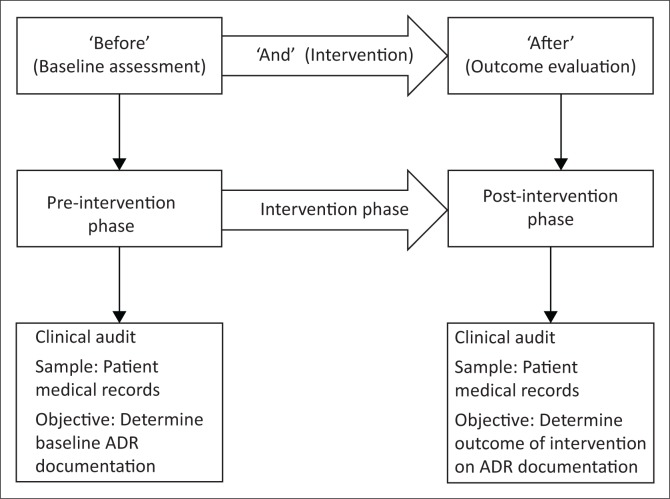
Research design illustrating the pre-intervention, intervention and post-intervention phases.

The study took the form of a quasi-experimental, uncontrolled ‘before and after’ intervention study^27^ (Figure 1). The study was empirical, focusing on quantitative data.^28,29,30^ A clinical audit of patient medical records was performed prior to and following the educational intervention. The pre-intervention phase of 1 month was designed to determine the baseline level of ADR documentation at the research site. The intervention phase followed during which the educational intervention was implemented over a period of 1 month. The post-intervention phase was designed to evaluate the impact of the intervention, 3 months after implementation, and thus the study design can be viewed as outcome evaluation research.^28^ The outcome to be evaluated was whether there was a change in the extent and frequency of ADR documentation after the educational intervention.

#### Setting

The study took place in an acute care, public sector psychiatric facility in the Eastern Cape, South Africa.

#### Study population and sampling strategy

Two populations were included in the study, namely the HCPs and the patient medical records.

The population targeted for the intervention phase consisted of the medical doctors and professional nurses employed at the study site. A purposive, total population sampling technique was employed, as the overall population of HCPs involved in documentation was relatively small. The expected sample size consisted of the HCPs employed at the study site at the start of the study, that is, 11 medical doctors and 71 professional nurses.

In order to assess the extent of ADR documentation prior to the intervention and the impact of the intervention, medical records of patients admitted to the 163-bed facility were analysed (i.e. the second population). Medical records for patients were drawn from all wards, so that the sample population ranged from patients who were acutely ill to those who were stable and nearing discharge. A non-probability convenience sampling technique was employed in order to review as many medical records as possible in a limited time period. The following inclusion criteria were applied:

- a current admission
- on treatment with an antipsychotic drug
- over the age of 18 years.

Patients with co-morbid disease states, and thus on treatment with additional medications, were included. A purpose-designed clinical audit data collection form was used to retrieve the relevant data from the paper-based patient medical records during the pre- and post-intervention phases. The clinical audit data collection form was piloted before the pre-intervention data collection phase, by auditing ten patient medical records; and after subsequent revision, a second batch of ten records were audited, after which the form was accepted.

#### Format of the intervention

The intervention consisted of the provision of education to medical doctors and professional nurses at the study site, as well as the insertion of ADR documentation forms (the intervention tool) in patient medical records. The educational content was developed and verbally presented by the primary investigator to the target audience of HCPs, using a presentation style format. HCPs were also provided with a printed version of the presentation slides for future reference purposes. The presentation content focused on the pharmacology of the antipsychotic drugs, signs and symptoms of antipsychotic ADRs and pharmacovigilance. HCPs also received education on the need to document symptoms or complaints, even if uncertain whether a symptom was drug-induced or not. The profession-specific training session was repeated on several occasions for each group of HCPs (medical doctors and professional nurses). The repetition was necessary as this ensured that all HCPs received training and thus accounted for staff absences arising from shift changes or illness or annual leave: the presentation was repeated three times for the professional nurses and twice for the medical doctors. A total of 11 medical doctors and 71 professional nurses were trained.

The ADR documentation form had two sections: the first section was used by the HCPs for the sole purpose of documentation of ADRs and included essential information, such as date, description and documenting professional. The second section served as a visual reminder of ADRs commonly associated with the antipsychotic drugs. The intervention tool (ADR documentation form) was developed by the principal investigator in collaboration with the chief psychiatrist and nursing manager, and subsequently tested and validated in a pilot study, prior to approval by the acting hospital manager at the research site. Once the HCP training had been completed, the intervention tool was introduced by inserting the printed forms in the patient medical records.

## Data collection

The pre-intervention clinical audit included 102 patient medical records, reviewed over a period of 1 month, whilst the post-intervention audit consisted of a separate sample of 102 patient medical records, collected over a period of 2 months. Because of patient turnover in the period of 7 months separating the before and after phases of the study, the patient medical records used in the pre-intervention audit were obtained from different patients compared to the post-intervention audit. This was verified by cross-checking the medical record number which is patient-specific.

Data were collected by conducting a review of patient medical records using the purpose-designed clinical audit data collection form. The following data were collected:

- patient demographic information
- diagnosis
- antipsychotic drug use
- details of documented comments in the clinical notes regarding observed or patient-identified signs or symptoms that could be linked to a possible ADR
- the category of HCP responsible for documentation.

## Data analysis

For the purpose of this study, ‘ADR documentation’ was defined as the documentation of symptoms experienced by the patient which were identified as likely to be related to antipsychotic drug use. ADR documentation included the term ‘side effect’ or ‘ADR’, and often mentioned the implicated drug. The ADR documentation in the patient medical records was subcategorised into two groups, based on the presence or absence of an ADR, meaning that the documentation provided evidence that the patients’ symptoms had been assessed, and the presence or absence of possible antipsychotic-induced ADRs had been documented. Documentation that records the absence of an ADR is useful for patient monitoring purposes, as the patient medical record then indicates when an ADR previously experienced by a patient is no longer present.

Data were captured into a spreadsheet using Microsoft Excel® and subsequently analysed using Statistica^®^. Descriptive statistical analysis was performed, and data were reported in the form of mean ± standard deviation, using frequencies and percentages. Inferential statistical analysis utilised Pearson’s Chi-square tests and Student’s *t*-test to compare pre- and post-intervention data. A significance level of 0.05 was used.

## Ethical considerations

Ethical approval was obtained from the Nelson Mandela Metropolitan University Research and Ethics Committee (Human), (Ethics Approval Number H13-HEA-PHA-005), the research site (hospital manager) and the province (Eastern Cape Department of Health). No patient names or file numbers were recorded as unique identifiers were used, and patient confidentiality was maintained at all times. The participating HCPs provided signed written informed consent to participate in the educational intervention, and unique identifiers were assigned to ensure participants’ confidentiality and anonymity.

## Results

### Patient demographics

The majority of patients were men, with 71.6% (73; *n* = 102) in the pre-intervention group and 79.4% (81; *n* = 102) in the post-intervention group. The mean age of the patients in the pre-intervention group was 35.61 ± 13.30 years, ranging from 18 to 76 years, and in the post-intervention group, was 36.55 ± 12.49 years, ranging from 20 to 69 years. No statistically significant difference was found in the gender distribution (Pearson Chi-square *p* = 0.192) or the age distribution (Student’s *t*-test *p* = 0.443) in the pre-intervention and post-intervention groups.

In the pre-intervention phase, 16 distinct diagnoses were recorded in the 102 patient medical records, with schizophrenia (38.24%; *n* = 39) being the most frequently recorded diagnosis, followed by psychosis secondary to general medical condition and substance-induced psychosis (11.76%; *n* = 12). Psychotic disorders were more frequently diagnosed than mood disorders, although of the mood disorders, bipolar type I (7.84%; *n* = 8), schizoaffective disorder (4.90%; *n* = 5) and schizoaffective disorder (bipolar type) (4.90%; *n* = 5) were most frequently diagnosed.

The post-intervention group was found to have a wider variety of diagnoses, with 26 distinct diagnoses being identified. The most frequently diagnosed condition was again schizophrenia (31.37%; *n* = 32), followed by substance-induced psychosis (14.70%; *n* = 15). The third most diagnosed condition was psychosis secondary to general medical condition (8.82%; *n* = 9). Psychotic disorders were again more prevalent than mood disorders. There was a statistically significant difference (Pearson’s Chi-square *p* = 0.004) in the diagnoses identified in the pre-intervention and post-intervention phases.

### Usage trends of antipsychotic drugs pre- and post-intervention

**FIGURE 2:**
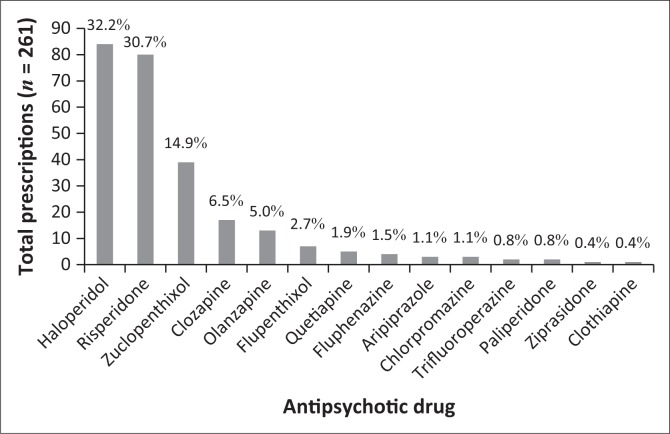
Pre-intervention usage trends of antipsychotic drugs.

**FIGURE 3:**
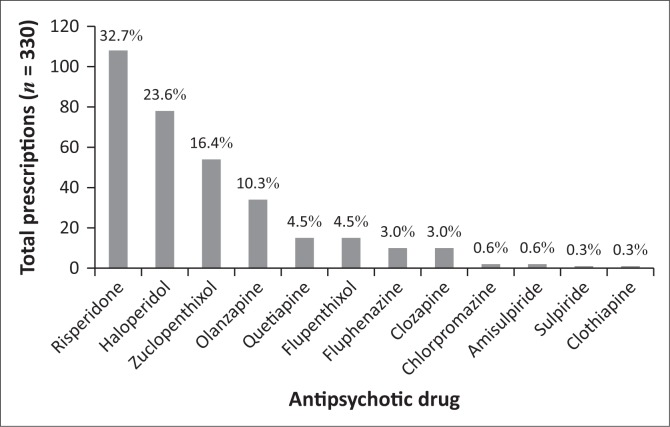
Post-intervention usage trends of antipsychotic drugs.

The clinical audit of patient medical records revealed that 14 different antipsychotic agents were prescribed during the pre-intervention phase, across a total of 261 prescriptions. Haloperidol was found to be the most frequently prescribed antipsychotic agent (32.2%; *n* = 84), followed by risperidone (30.7%; *n* = 80) and zuclopenthixol (14.9%; *n* = 39) (Figure 2). During the post-intervention phase, 12 different antipsychotic agents were prescribed across a total of 330 prescriptions. The most frequently prescribed drug in the post-intervention phase was risperidone (32.7%; *n* = 108), followed by haloperidol (23.6%; *n* = 78) and zuclopenthixol (14.9%; *n* = 39) as the most frequently prescribed long acting formulation (Figure 3). No statistically significant difference was noted in the overall frequency of antipsychotic prescription (Student’s *t*-test *p* = 0.510).

### Documentation of suspected antipsychotic-induced adverse drug reactions pre- and post-intervention

#### Prevalence of adverse drug reaction documentation

Acceptable instances of documentation of antipsychotic-induced ADRs included mention of the symptom or sign experienced, and may include the implicated antipsychotic drug. Patient medical records were first divided into records with evidence of ADR documentation and records lacking evidence of documentation, in order to describe the prevalence of ADR documentation (Table 1). There was a statistically significant increase in the number of patient medical records with ADR documentation in the post-intervention phase, with 16 more instances of documentation (Pearson’s Chi-square *p* < 0.05).

**TABLE 1:** Prevalence of documentation of adverse drug reaction in patient medical notes pre- and post-intervention.

Presence of documentation†	Pre-intervention (*n* = 102)	Post-intervention (*n* = 102)
No ADR documentation‡	36	20
ADR documentation§	66	82

ADR, adverse drug reaction.

† , Pre- versus post-intervention: Pearson’s Chi-square *p* < 0.05;

‡ , No evidence of documentation found;

§ , Evidence of documentation mentioning signs or symptoms suggestive of an antipsychotic-induced ADR.

Further examination of the patient medical records that contained evidence of ADR documentation (pre-intervention: *n* = 66; post-intervention: *n* = 82) revealed 186 instances of documentation pre-intervention (Table 2) compared to 351 instances post-intervention. A statistically significant difference was found in the number of instances of documentation reporting on the absence or presence of ADRs when the pre- and post-intervention results were compared (Pearson’s Chi-square *p* < 0.05). Surprisingly, only two instances of documentation post-intervention were recorded on the actual ADR documentation form (intervention tool).

**TABLE 2:** Frequency of type of adverse drug reaction documentation in patient medical records with evidence of adverse drug reaction documentation.

Type of documentation†	Pre-intervention % (*n* = 186)	Post-intervention % (*n* = 351)
Documentation noted ‘Absence of ADR’ ‡	37.63%	27.35%
	(70 instances)	(96 instances)
Documentation noted ‘Presence of ADR’ §	62.37%	72.65%
	(116 instances)	(255 instances)

ADR, adverse drug reaction.

† , ADR absence versus ADR presence: Pre-intervention compared to post-intervention: Pearson’s Chi-square *p* < 0.05;

‡ , Absence of ADR: Evidence of documentation noting the absence of any suspected antipsychotic-induced ADRs;

§ , Presence of ADR: Evidence of documentation noting the presence of suspected antipsychotic-induced ADRs.

When the type of HCP involved in documenting was analysed, it was found that medical doctors documented 77 instances of ADR documentation in the pre-intervention phase and 219 instances of documentation post-intervention. The professional nurses recorded 109 instances in pre-intervention phase and 132 instances in post-intervention.

#### Type of suspected antipsychotic induced adverse drug reactions

Before discussing the ADRs linked to the antipsychotic drugs (Table 3), it must be noted that instances of documentation recorded as an ADR were those suspected by HCPs to be antipsychotic-induced. It is thus possible that some suspected ADRs could have been because of other pharmacological therapy (e.g. sedation can be caused by multiple drugs), but the accuracy of the documentation was not verified as part of this study.

**TABLE 3:** Documentation of suspected antipsychotic-related adverse drug reactions, including multiple instances of the same adverse drug reaction in individual patients.

ADR documented	Pre-intervention % (*n* = 116)	Post-intervention % (*n* = 255)
Reversible EPSE	66.38	65.49
Sedation	14.66	12.55
Irreversible EPSE	4.31	5.49
Headache	2.59	-
Oedema	2.59	-
General side effect noted	1.72	0.39
Weakness	1.72	-
Hypersalivation	0.86	6.67
Orthostatic hypotension	0.86	2.34
Loss of libido	0.86	-
Metabolic side effects	0.86	-
Diarrhoea	0.86	-
Fever	0.86	-
Insomnia	0.86	-
Cardiotoxicity	-	0.39
Constipation	-	0.39
Oculogyric crisis (EPSE)	-	0.78
EPSE improved	-	1.96
Urinary retention	-	0.39
Hypoglycaemia	-	0.39
Tachycardia	-	1.57
Nausea/vomiting	-	0.39
Galactorrhoea	-	0.78

ADR, adverse drug reaction; EPSE, extrapyramidal side effects.

A total of 255 instances of the type of ADRs were documented in the post-intervention phase compared to 116 in the pre-intervention phase. This represents a 2.1-fold increase in the rate of documentation after the intervention, but these results include duplicate instances of documentation. Duplicates may include cases where more than one HCP has documented the same ADR in a different place in the patient medical record or where the same ADR was documented in the same patient over a number of consecutive days.

After exclusion of duplicate instances of documentation (Table 4), 66 instances of documentation during the pre-intervention phase were identified compared to 105 instances in the post-intervention phase. During the pre-intervention phase, the most frequently identified form of ADR documentation was reversible EPSE (56.06%; *n* = 37), followed by sedation (15.15%; *n* = 10). During the post-intervention phase, the results were similar, with reversible EPSE again being the most frequently identified ADR (48.57%; *n* = 51) and sedation as the second most frequently identified ADR (18.10%; *n* = 19). Hypersalivation was far more frequently identified in the post-intervention phase as 12.38% of all documentation (*n* = 13), compared to the pre-intervention phase (1.52%; *n* = 1). Several ADRs not identified at all during the pre-intervention phase were recognised in the post-intervention phase, including: ‘EPSE improved’ (4.76%; *n* = 5), tachycardia (1.90%; *n* = 2), urinary retention, constipation, oculogyric crisis, hypoglycaemia, nausea or vomiting and galactorrhoea (0.95%; *n* = 1).

**TABLE 4:** Documentation of adverse drug reactions where one instance represents one patient experiencing that particular adverse drug reaction (i.e. with duplication removed).

ADR documented	Pre-intervention %	Post-intervention %
	(*n* = 66)	(*n* = 105)
Reversible EPSE	56.06	48.57
Sedation	15.15	18.10
Irreversible EPSE	6.06	2.86
Headache	4.55	-
General side effect noted	3.03	0.95
Oedema	3.03	-
Hypersalivation	1.52	12.38
Orthostatic hypotension	1.52	3.81
Metabolic side effects	1.52	-
Loss of libido	1.52	-
Weakness	1.52	-
Fever	1.52	-
Insomnia	1.52	-
Diarrhoea	1.52	-
Cardiotoxicity	-	0.95
Constipation	-	0.95
Oculogyric crisis (EPSE)	-	0.95
EPSE improved	-	4.76
Urinary retention	-	0.95
Hypoglycaemia	-	0.95
Tachycardia	-	1.90
Nausea/vomiting	-	0.95
Galactorrhoea	-	0.95

ADR, adverse drug reaction; EPSE, extrapyramidal side effects.

## Discussion

The aim of this research was to determine whether the educational intervention had any impact on the extent and frequency of documentation of antipsychotic-induced ADRs. Table 1 provides evidence of increased ADR documentation in the patient medical records, suggesting that HCPs were more conscious of the need to document following the intervention. However, this documentation should indicate the presence or absence of an ADR. Although Table 2 shows evidence of increased instances of documentation of the presence or absence of suspected antipsychotic-induced ADRs, one concern is that documentation reporting an absence of suspected ADR-symptoms could mean a lack of recognition of relevant symptoms by HCPs.

Following the analysis of the type of suspected antipsychotic ADRs, it was found that the most frequently experienced ADRs were those relating to EPSE, both reversible and irreversible. EPSE are ADRs most commonly experienced by patients receiving treatment with first-generation antipsychotics, as well as with some second-generation antipsychotics (particularly high dose risperidone).^10,31^ Iversen et al. found that more than 75% of antipsychotic users reported adverse effects.^32^ As the relationship between adverse effects and poor adherence, symptom relapse and hospitalisation is well documented,^15^ recognition and minimisation of antipsychotic-induced adverse effects should be seen as an essential goal of treatment. General practice in psychiatry is to initiate a patient on any first-generation antipsychotic as first line therapy, followed by any second-generation antipsychotic, excluding clozapine.^33^ Availability of medicines in South Africa’s public sector is guided by the Department of Health’s Essential Medicines List,^34^ and haloperidol would be the first-generation antipsychotic of choice, whilst risperidone is the second-generation antipsychotic of choice. For long-term treatment, a depot preparation is often used, with zuclopenthixol being the most affordable and readily available. This practice in the public healthcare sector in South Africa was reflected in the results, with the three aforementioned drugs making up the bulk of antipsychotics prescribed in both the pre- and post-intervention phases (Figures 2 and 3), and also links to the finding that EPSEs were the most frequently encountered antipsychotic-induced ADR. Of interest was the more frequent use of risperidone in the post-intervention group, possibly because of the wider range of diagnoses encountered in this phase. In both pre- and post-intervention phases, the antipsychotic agents, which are less well known, more difficult to obtain or reserved for refractory cases, were less frequently prescribed. Similar findings were reported in the study where almost three-quarters of patients (74%, *n* = 169) admitted to an acute psychiatric hospital in the Eastern Cape were initiated on haloperidol, and 34% were subsequently switched to a second-generation antipsychotic during the admission period, because of EPSE (63%) or lack of efficacy (19%).^35^

In the current research, sedation was another ADR identified frequently in both phases. Sedation is associated with many of the antipsychotic drugs, and as such, its relative high frequency was expected, based on prescribing practices at the facility. In general, when the types of ADRs were analysed, all ADRs documented in their relative frequency were in line with the prescribing patterns of these drugs at the research site. The increased number of ADR types which were documented post-intervention implies that HCPs had a greater awareness of antipsychotic-induced ADRs, presumably because of the educational content of the intervention, and the section describing antipsychotic-induced ADRs in the ADR documentation form which was included in the patient medical records.

Nurses have been shown to lack knowledge in the area of ADRs and what signs and symptoms should be reported or documented,^36^ and thus require additional support and guidance. Peusschers et al. also reported a lack of documentation by nurses of potential adverse effects for psychotropic medications^23^ with less than half of the documented medication changes including the rationale. The authors highlighted that these omissions provide potential sources of medication errors. Similarly, in Ireland, only 14% of patient records were found to have documentation on EPSEs, but an educational intervention targeting medical doctors improved the incidence of documentation to 42%.^37^

Of interest in the current study was the finding that the intervention tool was only utilised by two medical doctors for ADR documentation purposes; therefore, the success of the intervention appears to be based on the educational sessions, which appear to have increased the awareness of the need to document and, possibly, improve the level of knowledge of the types of antipsychotic-induced ADRs that can occur.

Thus, it can be concluded that the educational intervention had a positive impact on the extent and frequency of documentation of antipsychotic-related ADRs, with an increase in the number of patient medical records containing some form of ADR documentation; an increase in the frequency of documentation of antipsychotic-related ADRs (including and excluding duplicate entries); and a greater range of ADR types identified. This suggests that educating the HCPs led to a greater understanding of the types of ADRs which may be induced by antipsychotic drugs. In addition, the increase in frequency of documentation, particularly of duplicate documentation, could imply a better understanding of the purpose of and need for documentation of all ADRs, particularly those which noticeably impact on patient quality of life (such as reversible and irreversible EPSE).

## Limitations and recommendations

This study was limited by the small sample size, as the data were collected from only one research site. In order to determine the overall effectiveness of an educational intervention on the parameters of documentation of ADRs, larger sample sizes from a greater number of sites would need to be used. One concern with uncontrolled before and after study designs is the possibility that the results may overestimate the effects of the intervention. Further research is needed to investigate the long-term sustainability of the educational intervention, particularly in the context of staff turnover. A closer examination of the usefulness of the ADR documentation tool as a means of improving documentation is also indicated. In addition, there was a degree of uncertainty in identifying which ADRs were specifically linked to antipsychotic drugs, as this was generally determined by the staff members’ understanding of antipsychotic-related ADRs. A final limitation of the study was the absence of electronic patient records at the research site, as the inclusion of an electronic version of the ADR documentation form may have further improved the level of documentation.

## Conclusion

This research found that documentation of antipsychotic-related ADRs improved as a result of the educational intervention in a number of areas, particularly the frequency of documentation and the number of types of ADRs documented as being potentially associated with antipsychotic drugs. However, further research involving a larger sample is recommended to test the generalisability and long-term sustainability of the intervention within a broader context.
